# Editorial: Muscle-Tendon-Innervation Unit: Degeneration and Aging—Pathophysiological and Regeneration Mechanisms

**DOI:** 10.3389/fnagi.2016.00320

**Published:** 2016-12-26

**Authors:** Luciano Merlini, Cesare Faldini, Paolo Bonaldo

**Affiliations:** ^1^Department of Biomedical and Neuromotor Sciences, University of BolognaBologna, Italy; ^2^Department of Orthopedics, Istituto Ortopedico Rizzoli, University of BolognaBologna, Italy; ^3^Department of Molecular Medicine, University of PadovaPadova, Italy

**Keywords:** muscle, skeletal, tendons, nerve fibers, aging, degenerative diseases

This research topic brings together basic researchers and clinicians working in the area of neuroscience, aging, sarcopenia, and orthopedics in human and in animal models with the aim to accelerate our understanding of the mechanisms involved in aging and degeneration of the muscle-tendon unit and its innervation and to explore the therapeutic potential of different interventions.

Tendon injuries are the most common cause of chronic pain and temporary/permanent disability in both the world of sport and at work. Tendinopathy, once mostly associated with inflammation and called tendonitis, is now considered the result from an imbalance between protective/regenerative modifications and acute or chronic tendon overload. The treatment modalities of tendinopathy are very different and even conflicting and have in common a lack of scientific evidence. It is therefore important to investigate the basic mechanisms that have translational purposes.

Chen et al. observed in the skeletal muscle of rabbits fed a 2% cholesterol-enriched diet for 12 weeks the presence of abnormally enlarged endolysosomes containing accumulations of free cholesterol and multiple Alzheimer's disease (AD) marker proteins subject to misfolding and aggregation including Aβ, phosphorylated tau, and ubiquitin. These results suggest that elevated levels of plasma cholesterol can alter endolysosome structure and function promoting the development of AD-like pathological features in skeletal muscle, which might contribute to the development of skeletal muscle dysfunction in AD patients (Chen et al.).

Schulz-Schaeffer is proposing that camptocormia in Parkinson's disease (PD), a situation characterized by an anterior flexion of the spine, is related to proprioceptive dysregulation. Camptocormia occurs in several conditions including myopathies, myositis, dystonia, and is particularly frequent in PD. Besides central proprioceptive dysregulation of muscle tone like in PD-associated camptocormia, similar myopathological changes can be observed whether the alterations of the proprioceptive polysynaptic reflex arch occur at the level of the Golgi tendon organ, the dorsal root ganglia, or nerves by disk herniation or aging spondylotic changes (Schulz-Schaeffer).

Vasta et al. reviewed the biological processes involved in tendon healing including the synthesis of collagen and focusing in particular on the role of vascular and neuronal molecular pathways. Vascular endothelial growth factor (VEGF) has shown a key angiofibroblastic role in tendon healing both in animal models and *in vivo* in patients. Recent studies have enlightened the positive role of neurotransmitters like substance P and nitric oxide in the treatment of tendinopathy, supporting the hypothesis of a nerve-mediated dysregulation of tendon metabolism (Vasta et al.).

Yin et al. report that semaphorin 3A is a potent inhibitor of axonal outgrowth and vascular proliferation and has a negative regulation of the matrix metalloproteinases, which are involved in degradation of extracellular matrix proteins of disk. This suggests that semaphorin 3A may act as a potential target for low back pain (Yin et al.).

Rosso et al. reviewed the role of mechanical stimulation by means of extracorporeal shock wave (ESW) and pulsed electromagnetic fields (PEMF) for the treatment of tendinopathy and tendon regeneration. While preclinical and *in vitro* studies suggested effectiveness, the authors pointed out that there is still a lack of strong evidence of the efficacy of these modalities in the clinical settings, suggesting the need of further *in vivo* human studies to confirm the clinical efficacy of mechanical stimulation for the treatment of tendinopathies (Rosso et al.).

Parchi et al. reviewed the various applications of nanoparticles for tendon healing and regeneration: labeling tendon stem cells, acting as carrier for gene therapy and drug delivery, allowing the construction of a new generation of bioactive scaffolds, and modulating the cellular and extracellular matrix response. In their paper, the authors highlighted and summarized the most recent advances and results (Parchi et al.).

Frizziero et al. reviewed the most recent clinical and experimental studies regarding the consequences of detraining in tendon mechanobiology. Overall, the *in vitro* and *in vivo* models showed, after detraining, noticeable alterations of tissue structural organization and of mechanical properties. Although clinical studies showed more variable results, the authors suggest that after a period of sudden detraining physical activity including rehabilitation should be restarted with caution (Frizziero et al.).

Giusti and Pepe described the organization of elastic fibers in tendon and particularly of fibrillin 1 and fibrillin 2 in some animal models and in humans. They review the clinical manifestations of monogenic disorders due to mutations in the genes of fibrillin 1 (Marfan syndrome) and fibrillin 2 (Congenital contractural arachnodactyly), which may cause a combination of joint hypermobility and contractures (Giusti and Pepe).

Popov et al. studied the aged related phenotype of tendon stem/progenitor cells (TSPCs) and its effect on tendon maintenance and healing. Their results showed a significant down regulation of the ephrin receptors EphA4, EphB2, and EphB4 and ligand EFNB1 in aged TCPC. These novel data suggest that decreased expression of ephrin receptors during tendon aging and degeneration limits the establishment of appropriate cell–cell interactions between TSPC and significantly diminishes their proliferation, motility and actin turnover (Popov et al.).

Raz et al. investigated age-associated changes in shoulder muscle pathology combining radiological and histological procedures. They found that patterns of age-associated muscle degeneration were similar between individuals with and without muscle tears. Furthermore, they found that torn rotator cuff muscles display tissue hallmarks of muscle aging, including fatty infiltration, increase in extra-cellular matrix, and loss of slow oxidative myofibers. In contrast, the teres minor exhibited healthy muscle features suggesting that teres minor could represent an aging-resilient muscle (Raz et al.).

Salini et al. compared the efficacy of platelet rich plasma (PRP) therapy in young and elderly subjects suffering for Achilles tendinopathy. They found that PRP is less effective in aged people, suggesting that this can be ascribed to several biochemical and biomechanical differences documented in tendons of young and elderly subjects with the latter showing reduced number and functionality of tenocytes and tenoblasts, which become more evident in the long-term tissue healing (Salini et al.).

Sardone et al. studied a peroneal tendon biopsy and tenocyte culture of a patient with Ullrich congenital muscular dystrophy (UCMD) due to compound heterozygous mutations in the *COL6A2* gene. They found irregular profile and reduced mean diameter of tendon fibrils, and abnormal accumulation of collagen VI and altered distribution of collagen I and fibronectin. In tenocyte culture, collagen VI web formation and cell surface association were severely impaired and metalloproteinase MMP-2 was increased in the conditioned medium. Altogether, these new data indicate that collagen VI deficiency may influence the organization of UCMD tendon matrix, resulting in dysfunctional fibrillogenesis (Sardone et al.).

Finally, we realize that this collection of articles and reviews cannot exhaust all lines of research related to the biology and the pathology of the muscle-tendon complex and its innervation. These articles, however, outline the main pathogenetic mechanisms and thus the possible rehabilitation therapeutic interventions to counteract the aging process of the nerve-muscle-tendon unit. We hope that the items of this collection will be a stimulus for future progress toward a niche of research with great expectations for aging well.

## Author contributions

All authors listed, have made substantial, direct and intellectual contribution to the work, and approved it for publication.

### Conflict of interest statement

The authors declare that the research was conducted in the absence of any commercial or financial relationships that could be construed as a potential conflict of interest.

